# Supplementary material for: Risk map development for soil-transmitted helminth infections in Argentina
Source: PLoS Negl Trop Dis. 2020 Feb 10;14(2):e0008000. doi: 10.1371/journal.pntd.0008000 (PMC7010237; doi:10.1371/journal.pntd.0008000)
Supplement: Attachment — Submitted filename: Response to reviewers.docx [file pntd.0008000.s001.docx]

**Point-by-point letter of response so reviewers.**

First of all, we would like to thank the reviewers for their time and valuable comments/suggestions to improve our manuscript.

Responses to Reviewers Questions

**Key Review Criteria Required for Acceptance?**

As you describe the new analyses required for acceptance, please consider the following:

**Methods**

-Are the objectives of the study clearly articulated with a clear testable hypothesis stated?

-Is the study design appropriate to address the stated objectives?

-Is the population clearly described and appropriate for the hypothesis being tested?

-Is the sample size sufficient to ensure adequate power to address the hypothesis being tested?

-Were correct statistical analysis used to support conclusions?

-Are there concerns about ethical or regulatory requirements being met?

Reviewer #1: The method used in not completely new but very clearly explained . The verification of the results of the model with real data is an important aspect of the paper.

Reviewer #2: Limitations of the methods have not been addressed.

The limitations of the methods have been added to the discussion section.

--------------------

**Results**

-Does the analysis presented match the analysis plan?

-Are the results clearly and completely presented?

-Are the figures (Tables, Images) of sufficient quality for clarity?

Reviewer #1: In the results session (Table 4) the authors are comparing the 3 indexes with the real results; in my opinion this is not ideal because is difficult to understand when the model predicted well and where failed to predict.

For example, San Caytano SEC=0.44; EC=0.7 SC=1; is not clear to me what is the prediction from the model in term of STH prevalence? One index (SEC) is the lower of the list in the list one other is the higher (SC).

I think that the authors, in addition to “SEC Risk” “EC Risk” and “SC risk”, should also provide a composite index that predicts a unique STH prevalence.

I do not think the thee indexes are equally important in predicting the STH prevalence and therefore the 3 indexes can be given different weight in the formula.

Then in table 4 I would provide for each city a “predicted STH prevalence” (low - moderate - high - very high) and a “real STH prevalence” (low - moderate - high - very high) This will make very simple to evaluate if the prediction modeled were verified in reality.

Thank you for your comments, this makes it clear to us that we were not very successful in explaining out method. When we calculated the different risk factors (socioeconomic, environmental and soil characteristics) that we believe are associated with the presence of STH and various studies have shown this association, we also think that there are some indexes that are more important than other, or put in other words, we believe that some HAVE to be present in order for transmission to occur, but others not necessarily. For example, in a previous study (Periago et al., 2018), the environmental conditions and socioeconomic conditions were met, but because the soil conditions were NOT met, there was a lack of STH. On the other hand, you might encounter a scenario were the environmental and soil conditions are met but not the socioeconomic ones, therefore although the soil might be contaminated if a person lives in a house with water and sanitation and practices hygiene and wears shoes, they will not become infected. Therefore, in reality we know that all three would need to be present but there is a lack of studies that statistically prove that one is more important over the other and how much more important, 2, 3 or 4 times? This is why in the end, we decided not to formulate an equation since we didn´t have enough information to decide if we should add the 3 factors, multiply them or what…

So what we have decided to do, based on your suggestion, is to formulate an objective rule using machine learning concepts, specifically decision tree models. This model was added to the manuscript in the methodology, results (Table 4) and discussion and it predicted the prevalence class of each city of the 20 prevalence studies included in order to be able to compare it with the “real” prevalence obtained through fecal analysis of the samples.

The authors attempt to do what I suggested in figure 9 but in my opinion this is not clear which color correspond to high risk? There is no legend clarifying this the color-code triangle in my opinion is not helpful because it gives only the proportion of the different indexes. Which color will be a city A in which all the 3 indexes are predicting consistently high prevalence? And which color in a city B in which all the indexes predict consistently low prevalence? Probably with the method used by the author the two cities will have similar color…

The idea of how much each of these factors contributes in any given place is the result of Figure 9. All the values entered pass through the RGB system which have values between 0-255. So if the values of all 3 characteristics is (0,0,0), the dot will be black, if the values are (255,0,0), the dot will be red, if the values are (0,255,0), the dot is green and if the values are (0,0,255), the dot will be blue. Finally, if for example the maximum value of all three components are entered (255,255,255), the dot will be white.

Here is an image to illustrate (where A is high – “alto” in Spanish, M is Medium and B is low – “Bajo” in Spanish):

In other words, if all three of these risks are high, the dot is light colored and all the risks are low, then the dot is dark colored. Then, if you mix red and blue you get more purple, green and red you get more orange, etc. This way you know the magnitude of each type of risk in each point. We believe it’s an innovative way to view the interaction between different types of characteristics. This is reflected in the chromatic table above, the A city will have a clear color (near to white) and the B city will have a dark color (near to black).

Reviewer #2: The study lacks uncertainty measures.

This is tied to the limitations of the method and in reality there are no uncertainty measures in this type of analysis, although they might be the sum of the uncertainty measures of each method used. This was added to the discussion. Additionally, in the text below, we answer your specific question on the error of the variables.

--------------------

**Conclusions**


-Are the conclusions supported by the data presented?

-Are the limitations of analysis clearly described?

-Do the authors discuss how these data can be helpful to advance our understanding of the topic under study?

-Is public health relevance addressed?

Reviewer #1: conclusions are OK, but the method should be improved as suggested.

OK, answered above.

Reviewer #2: Validation methods should be discussed further and limitations should be disclosed.

These have been added as suggested.

--------------------

**Editorial and Data Presentation Modifications?**

Use this section for editorial suggestions as well as relatively minor modifications of existing data that would enhance clarity. If the only modifications needed are minor and/or editorial, you may wish to recommend “Minor Revision” or “Accept”.

Reviewer #1: Minor comments:

• Page 8 I would use “Preventive chemotherapy” instead of “Mass Drug Administration” because the Mass Drug Administration is a form of Preventive chemotherapy that is aiming to cover the entire population, while in the case of STH only groups at risk are recommended for treatment.

Thank you for noting that, it has been modified as suggested.

• After the first citation please refers to the STH species as A. lumbricoides or T. trichiura and not as Ascaris or Trichuris

Thank you for noting that, it has been modified as suggested.

Reviewer #2: (No Response)

--------------------

**Summary and General Comments**

Use this section to provide overall comments, discuss strengths/weaknesses of the study, novelty, significance, general execution and scholarship. You may also include additional comments for the author, including concerns about dual publication, research ethics, or publication ethics. If requesting major revision, please articulate the new experiments that are needed.

Reviewer #1: The article address a relevant issue in the field of NTD, is well written and in my opinion merits publication with minor revisions

Reviewer #2: Authors are presenting a risk map of soil-transmitted helminths (STH) in Argentina developed using an analytical hierarchy process were 20 experts in the field decided the importance of socioeconomic, environmental (climates and altitude), and soil variables on the development of STHs infections. Although the study is addressing a gap in knowledge, they should improve their discussion and present uncertainty measures. Some recommendations based on sections since the paper lacked page numbers or lines:

Introduction
Paragraph 1 and 2 should be together, they are talking about the same thing. If you want to split them, consider separating them in ‘For control…’

Thank you for the suggestion, these paragraphs were put together.

Paragraph 2: In the last sentence ‘it is not included’ affirmation is referring to WHO guidelines for deworming?

That it right, but this sentence was removed since it seemed a bit redundant. Thank you.

Last paragraph: Refer to table 2 where all the references of STHs studies in Argentina are presented.

This reference to the table was added.

Methods:
Since it is the only equation, I would suggest eliminating the number inside square brackets (i.e., [1]).

Thank you, the brackets and number were removed.

Data collection:

Worldclim does not offer soil variables, only climatic data for temperature and precipitation. Which pixel size (resolution) of worldclim variables was used? This is key since you will be having different values in your analysis if you are using 5-minute arc, 2.5-minute arc, etc.

There is not enough detail of the type of NDVI data collected. You should describe the resolution of the variables, the source of the images, and also the satellite from where the index was derived; it is different to use 500-meter biweekly resolution variables than 1 km monthly variables considering that you are using averages. Also, it is important to detail the source (NASA-Earth data?), the version (e.g., 006), and the satellite from where they were derived (Terra, Aqua, the products from both satellites?).

Please describe SERENA land cover variables briefly, are those a categorical product showing different classes of environments at percentages? What is the resolution of the altitude variable from SRTM?

Please provide a brief description for the diversity index developed for the cities studied.

Following the suggestion of the reviewer, a paragraph was added to the method section with information on the spatial resolution of NDVI, Serena´s land cover types and high resolution of Dem, among other details of the variables used.

Table 1:

Does the variable urban population should be % of urban population as in the main text?

Yes, thank you for noticing that, it was corrected.

Figures:
It is recommended that all the figures follow equal features, currently figure 1 and three have not frames but figure 2 and 4 have them. North arrow is different depending on the figures.
Also, consider adding Argentinean provinces in all maps (currently only in figures 8-9) and potentially label the provinces discussed in the main text.

Thank you for your suggestion, this really helps in the understanding of the study for those unfamiliar with the political map of Argentina. We have standardized the figures so that they all have the same features. The name of the provinces was labeled only in Figure XX since in other figures it would be hard to visualize.

Why Fig 6 (Environments) is presented as a continuous map while Figs 5 and 7 are presented as points? This question is relevant because the RGB map (Figs 8 and 9) is showing environments as point values again. Is Fig 6 interpolating values?

Add province names in Figs 9-11.

Continuous data is available for environmental data while only point data is available for the other two maps, in order for the methodology to be replicated, were decided to leave the environmental data as continuous since that is a map that is easy to replicate in other parts of the world, the other two maps are more country specific since not all countries have a census or availability of soil data. The data from Figure 6 is not interpolating values.

Figures 1-3 can be combined in one figure.

Figures 4-6 can be combined in one figure.

Figures 10-11 can be combined in one figure.

We understand that putting certain figure together would be better so for clarity we have combined figures 2, 3 and 4 in one (now Figure 2).

Moreover, we have modified Figure 1 (Diagram of the methodology) to include de decision tree generated through the use of machine learning and the prevalence data from the 20 studies included. Since we have added and used this methodology, we removed Figures 10 and 11 since and we added Figure 8 which reflects the Decision Tree Output.

We tried combining the other figures suggested but the resolution was too low for clear comprehension. In Figure 4 we have added the provinces in order to guide comprehension but did not include it in the resto of the figures since with the individual dots, it would be hard to see.

Results and discussion:

Would you be able to present uncertainty measures for your predictions? I am mentioning this because your model validated 15 over 20 localities where prevalence data is available, however, you applied your model to 3526 cities. Thus, it will be useful to address how good is your prediction by considering points without available information.

Consider also discussing limitations of your approach, at present you are not declaring any potential problem with your AHP including variable selection and expert opinion. Further, prevalence of the papers considered in this study for validation might also be biased considering their own limitations and diagnostic methods; this should be also explicit to assure a fair treatment of their analysis.

Error of the methods combined. Limitation of the methods have been added and the limitation on prevalence studies, including techniques used for diagnosis have been mentioned in the discussion.

The present study used environmental, sol and socioeconomic characteristics that were obtained from different sources and the precision for each one is analyzed below:

Socioeconomic variables – The origin of these variables is the 2010 National Census performed by the National Institute of Statistics and Census (INDEC) of Argentina [1]. If this survey is considered “ideal”, one should consider that all the homes have been surveyed, that all the questions were answered and that the questions themselves were all the correct ones in order to obtain the expected results. In this context, the data reported might be considered error free. Since it is expected that the situation was not “ideal” and since we don´t have the means of knowing/detecting which are the sources of the error, we have accessed the tables of errors included in the publication of the census itself [2]. From the analysis of this table, we can conclude that the variables included in our study (population with sanitation at home, % urban population, % population with access to safe drinking water, % population with computer, % population with overcrowding, unemployment rate) have a relative error under 5%.

Soil characteristics – Since these variables are categorical (Table 2), we have reasonably considered that the error could be estimated as inversely proportional to the quantity of classes. In other words, the inverse of the possible failures of categorization. Considering the relative weight of the variables, the variable of organic content is 3x more important that the rest, reason why we consider this as the reference variable of the analysis of error. This variable has 5 categories, therefore we consider the error to be of 20%, this will serve as the upper limit for the rest of the soil variables. Additionally, this variable has an intrinsic cartographic error that is not possible to know precisely but the one step category adopted above would cover this source of error.

Environmental characteristics – The origin of these variables are different studies (A land cover map of Latin America and the Caribbean in the framework of the SERENA project by Paula Blanco et al. [3], and Very high resolution interpolated climate surfaces of the global land areas by Robert Hijmans et al. [4]) whose data may be downloaded from the WorldClim – Global Climate Data repository [5]. The variable taken from SERENA is Land Cover Type, for Argentina, it reports an average quadratic error between the coverage reported and the one estimated by pixel with an average of 13.95 and standard deviation of 4.86. With respect to the rest of the environmental variables, taken from Hijmans et al. [4], the update of the original study “WorldClim 2: New 1-km spatial resolution climate surfaces for global land areas” by SE Fisks and RJ Hijman [6] where they report average quadratic error lower than 2, except for precipitation which is the variable with most uncertainty, with a reported error of 21.29 (which means around 5% relative error). As with the soil variables, the precipitation variable is the one with the most weight in the mode and therefore it is the error taken as the upper limit for the rest of the environmental variables.

References:

[1] <https://www.indec.gob.ar/ftp/cuadros/poblacion/censo2010_tomo1.pdf>
[2] <https://www.indec.gob.ar/ftp/cuadros/menusuperior/eph/EPH_errores_muestreo_3t2014.pdf>
[3] <https://reader.elsevier.com/reader/sd/pii/S0034425713000035?token=E3B43AE9CEE12C2E8DE53BDE51CDCFF1B31A76558A0CBA2F9E1C4788473DA57CBE8DB73A0F0DBB09108CAF9E1697E402>
[4] <https://rmets.onlinelibrary.wiley.com/doi/epdf/10.1002/joc.1276>
[5] <http://worldclim.org/bioclim>
[6] <https://rmets.onlinelibrary.wiley.com/doi/full/10.1002/joc.5086>

Others:

Consider that the species status of Ascaris lumbricoides and A. suum is being currently discussed posing a potential zoonotic feature of the genus Ascaris spp. (Review: https://doi.org/10.1017/S0022149X18000160 and <https://doi.org/10.1186/1756-3305-5-42>)

Thank you very much for this interesting topic, although I understand it is still under discussion and in the studies included for validation, Ascaris spp. Was not the most prevalence species of STH found. This seems to be the case in in Argentina, much more presence of hookworm and Strongyloides than Ascaris spp. and Trichura spp.

Review references, for example, reference 3 starts with a year. Some references are mentioning the name of the article twice.

Thank you, the references have been reviewed accordingly.

--------------------
PLOS authors have the option to publish the peer review history of their article ([what does this mean?](https://journals.plos.org/plosntds/s/editorial-and-peer-review-process#loc-peer-review-history)). If published, this will include your full peer review and any attached files.

If you choose “no”, your identity will remain anonymous but your review may still be made public.

**Do you want your identity to be public for this peer review?** For information about this choice, including consent withdrawal, please see our [Privacy Policy](https://www.plos.org/privacy-policy).

Reviewer #1: Yes: Antonio Montresor

Reviewer #2: No
