# Supplementary material for: Risk map development for soil-transmitted helminth infections in Argentina
Source: PLoS Negl Trop Dis. 2020 Feb 10;14(2):e0008000. doi: 10.1371/journal.pntd.0008000 (PMC7010237; doi:10.1371/journal.pntd.0008000)
Supplement: Attachment — Submitted filename: Response to reviewers.docx [file pntd.0008000.s002.docx]

First of all, we would like to thank the reviewer for their comments and suggestions of improvement. Please find below the point-by point response.

Response to reviewer:

Line 105: Eigenvalue matrix – what does this mean? Do the Authors mean the eigenvalue of a matrix? I suggest using a more accessible description. This description also appears on line 190. It will not be clear to the readership what this means.

Changes were made accordingly in line 103 and the legend of Figure 2.

Line 107: change lineal to linear

This was changed accordingly.

Line 114, 122 and elsewhere: “data” is a plural noun, therefore the text should read “data are”

This was changed throughout the manuscript.

Line 125: Could the authors clarify what “NDVI products” means?

This was added in lines 120 to 124.

Line 128: I believe the word “approach” should read “index”.

This was changed accordingly.

Lines 125-139: In the interests of clarity it would be helpful if the Authors could give the resolution of the different forms of remotely sensed data in standard and consistent units.

This was modified accordingly. All units were set in arc second and its equivalent approximation in meters.

Line 154: delete “Therefore”

This was modified as suggested.

Line 173: Could the Authors please clarify or give further detail on how model accuracy was quantified? The current wording, “Measure of model accuracy was obtained comparing the expected statistic of a variable distribution of the error with the four categories of ordinal data and the observed error value with its p-value.” is not clear.

The phrase was modified in lines 169 to 172. The error is obtained considering the incorrect assignments over the correct ones in each risk category. It is computed as a contingency table but accounting for ordinal data (not categorical).

Lines 182- 184: Please use “percentage” rather than “%”

This was modified as suggested.

Lines 185-188: Please review the use of capital letters.

This was reviewed and modified accordingly.

The legends of Figures 2 to 7 are too brief and mainly consist of a title only. Please remember that the figure legend is supposed to give (brief) relevant information to allow a reader to understand the main message in isolation from the text. For example, in Figures 5, 6 and 7, the meaning of the points should be described. Are these populations?

These were all modified as suggested.

Figure 5. It is not entirely clear to me why soil-risk was not presented continuously (as environmental risk in Figure 4). Is this an issue with the availability of data or does it relate to where human populations are?

Yes, the issue is of data availability since the risk of soil was computed from the beginning only in the different localities since their origin is in vector format, and the information from raster images is not as smooth. Therefore, the use of a continuous visualization would not be appropriate in this particular case.

Line 240: I do not think the machine learning approach describe constitutes a “model validation”. It seems that the Authors have combine the environmental/socioeconomic risks with prevalence data to fit a decision tree model. This is fine but it is not validation. That would be testing the model predictions against prevalence data that were not used for training/fitting.

We agree with the reviewer that the machine learning approach is not a validation. Accordingly, we have changed the title of this section from “model validation” to “modeling”, In fact, in this section we include two parts, one is the very simple machine learning risk model and the second is the comparison with the data from the few field studies published in the literature. We only have data from 20 cities, so the set is too small to split it in training and test subsets. For this reason, we adopted the AHP methodology as the approach more than a statistical one. In our second version of the manuscript we included the ML approach following a reviewer´s suggestion.

Line 286: Can the Authors specify which STH species have been identified in Argentina?

This is sated in line 276. All 5 species of STH´s have been identified in Argentina.

Line 320: Again, I do not think the predictions have been “validated” – as the Authors state, the prevalence data used is key to informing the predictive power of the model but, as I understand, no data were withheld from training to be used for validation.

This is correct, we have replied above. In this section we are suggesting a methodology for a baseline STH study in Argentina, in order to guide the identification of survey areas.

Since the Authors have recent census data for Argentina, the manuscript would be strengthened by enumerating the population living in low, moderate and high risk (high prevalence) areas.

Through the use of the decision tree and the data from the national census, we calculated there are 247 very high risk localities divided in 5 provinces, 114 in Misiones, 54 in Formosa, 34 in Salta, 23 in Chaco and 22 in Corrientes). Most of these localities are small, with an average population of 1143 inhabitants. The total high risk population would be approximately 1,464,235 inhabitants. This information has been added to the results section, lines 256 to 260, and to the discussion section, lines 333 and 334.
